# Updating the Data on Malaria Vectors in Malaysia: Protocol for a Scoping Review

**DOI:** 10.2196/39798

**Published:** 2023-03-06

**Authors:** Rafidah Ali, Wan Najdah Wan Mohamad Ali, Perada Wilson Putit

**Affiliations:** 1 Institute for Public Health National Institutes of Health Selangor Malaysia; 2 Medical Entomology Unit Institute for Medical Research National Institutes of Health Selangor Malaysia; 3 Entomology and Pest Sector Ministry of Health, Malaysia Putrajaya Malaysia

**Keywords:** malaria vector, Anopheles, Malaysia, scoping review, malaria, vector, transmission, prevention, entomology, eliminating malaria

## Abstract

**Background:**

Malaria is still a public health threat. From 2015 to 2021, a total of 23,214 malaria cases were recorded in Malaysia. Thus, effective intervention and key entomological information are vital for interrupting or preventing malaria transmission. Therefore, the availability of malaria vector information is desperately needed.

**Objective:**

The objective of our study is to update the list of human and zoonotic malaria vectors in Malaysia. This work will include (1) the characterization of the key behavioral traits and breeding sites of malaria vectors and (2) the determination of new and potential malaria vectors in Malaysia. The findings of our scoping review will serve as decision-making evidence that stakeholders and decision makers can use to strengthen and intensify malaria surveillance in Malaysia.

**Methods:**

The scoping review will be conducted based on the following four electronic databases: Scopus, PubMed, Google Scholar, and Science Direct. A search strategy was conducted for articles published from database inception to March 2022. The criteria for article inclusion were any malaria vector–related studies conducted in Malaysia (with no time frame restrictions) and peer-reviewed studies. The PRISMA-ScR (Preferred Reporting Items for Systematic Reviews and Meta-Analyses Extension for Scoping Reviews) will be used to guide our systematic approach. Data from published research literature will be extracted by using a standardized data extraction framework, including the titles, abstracts, characteristics, and main findings of the included studies. To assess the risk of bias, articles will be screened independently by 2 reviewers, and a third reviewer will make the final decision if disagreements occur.

**Results:**

The study commenced in June 2021, and it is planned to be completed at end of 2022. As of early 2022, we identified 631 articles. After accessing and evaluating the articles, 48 were found to be eligible. Full-text screening will be conducted in mid-2022. The results of the scoping review will be published as an open-access article in a peer-reviewed journal.

**Conclusions:**

Our novel scoping review of malaria vectors in Malaysia will provide a comprehensive evidence summary of updated, relevant information. An understanding of the status of *Anopheles* as malaria vectors and the knowledge generated from the behavioral characteristics of malaria vectors are the key components in making effective interventions for eliminating malaria.

**International Registered Report Identifier (IRRID):**

DERR1-10.2196/39798

## Introduction

Malaria is still a public health threat. The World Health Organization (WHO) recorded a total of 241 million malaria cases and 627,000 malaria deaths worldwide in 2020 [1]. Further, Malaysia recorded 23,214 malaria cases from 2015 to 2021, of which 87% were zoonotic cases [1]. Malaysia is facing increasing cases of zoonotic malaria due to *Plasmodium knowlesi*. The number of *P knowlesi* cases increased from 1960 to more than 4000 between 2016 and 2018. This number declined slightly in 2019 and 2020 (to 3213 and 2609 cases, respectively), but *P knowlesi* infection still resulted in 6 and 5 deaths in 2019 and 2020, respectively [1].

Malaria is caused by parasites of the genus *Plasmodium*. These parasites harm not only humans but also other vertebrate species, including birds, reptiles, and mammals. *Plasmodium* species that naturally infect humans and cause malaria in large areas of the world were limited to the following four: *Plasmodium falciparum*, *Plasmodium vivax*, *Plasmodium malariae*, and *Plasmodium ovale* [2]. However, studies conducted by Cox-Singh et al [3], Piera et al [4], Siner et al [5], and Yek et al [6] confirmed that there is a fifth *Plasmodium* species responsible for zoonotic malaria—*Plasmodium knowlesi*.

Malaria is a vector-borne disease transmitted by *Anopheles* mosquitoes [7-9]. The WHO recorded 4500 species of mosquitoes worldwide in 34 genera from the family Culicidae, the order Diptera, the class Insecta, and the phylum Arthropoda [10]. Interestingly, only 70 species of anophelines were confirmed as malaria vectors around the world [10]. A total of 434 mosquito species have been recorded in Malaysia [11], including 75 species of *Anopheles* mosquitoes [12]. Previous studies recorded the following nine species of *Anopheles*, which were established as malaria vectors in Malaysia: *Anopheles balabacensis, Anopheles maculatus*, *Anopheles campestris*, *Anopheles sundaicus*, *Anopheles letifer*, *Anopheles donaldi*, *Anopheles dirus*, *Anopheles leucosphyrus*, and *Anopheles flavirostris* [12,13]. It is important to investigate potential malaria vectors when developing new technology for species complex identification. A potential malaria vector is defined by the vectorial capacity of a vector population to transmit malaria [14].

Malaysia is working toward eliminating malaria and preventing malaria re-establishment [15]. Effective entomological surveillance tools are the most important tools for interrupting malaria transmission [16-18], which can be achieved by designing a targeted control intervention based on the behavioral characteristics of malaria vectors [17,19-21]. For example, *Anopheles balabacensis* rests outdoors after feeding [22]. Therefore, vector control tools need to be developed for and applied on the walls of houses, tree trunks, bushes, or any possible resting places of *Anopheles balabacensis*, and existing outdoor vector control interventions should be strengthened. Our review will serve as a resource for informing entomologists, malaria personnel, and physicians about malaria vectors in Malaysia. Entomological information accelerates the process of malaria elimination by enhancing the impact and efficacy of the intervention needed [18]. The objectives of our study are to establish protocols for determining new and potential malaria vectors and to update the list of existing human and zoonotic malaria vectors in Malaysia.

## Methods

### Protocol Design

Our study will broadly cover the subject area—the nature of research activity on malaria vectors—in accordance with the 6-stage framework of Arksey and O’Malley [23], which was refined by Levac et al [24]. The six stages of the review process are as follows: (1) identifying the research question; (2) identifying relevant studies; (3) selecting studies; (4) charting the data; (5) collating, summarizing, and reporting the results; and (6) consulting with relevant stakeholders. The scoping review will adhere to the 22-item PRISMA-ScR (Preferred Reporting Items for Systematic Reviews and Meta-Analyses Extension for Scoping Review) checklist [25].

### Stage 1: Identifying the Research Questions

In order to develop and identify the research questions, the subject area—the nature of research activity on malaria vectors—was examined. Thus, the following research questions were identified: (1) what are the established malaria vectors in Malaysia and (2) what are the potential malaria vectors in Malaysia?

### Stage 2: Identifying Relevant Studies

A search strategy was conducted for articles published in scientific journals and peer-reviewed publications, using the following electronic databases: PubMed, Scopus, Google Scholar, and Science Direct. Relevant research websites, such as Portal Data Terbuka Malaysia [26], WHO websites, and Ministry of Health Virtual Library, were also searched.

Articles were identified by using Medical Subject Headings and keywords. Using a systematic approach, titles, abstracts, and keywords were searched, screened, and reviewed, and data were extracted. The search was not restricted by year or language. All selected search results were imported into Mendeley software (Elsevier) and Microsoft Excel spreadsheets (Microsoft Corporation) to manage references and duplicates.

### Stage 3: Study Selection

All entomological research conducted in Malaysia will be included in the study, including longitudinal studies, cross-sectional studies, observational studies, and descriptive reports. The literature search was only conducted for peer-reviewed articles published from database inception to March 2022. The exclusion criteria for the scoping review are gray literature, annual reports, and bulletins. First, inclusion and exclusion criteria will be used to determine the eligibility of articles based on their titles during the screening portion of the review process. Any articles with titles indicating that the research was conducted outside of Malaysia will be excluded. Second, titles and abstracts will be selected based on eligibility criteria. Only abstracts that fulfill the inclusion criteria will be further analyzed. Finally, full-text articles that are selected based on their abstracts will be reviewed and included in the study if they are considered significant and relevant studies.

### Stage 4: Charting the Data

The significant study characteristics of published research literature will be extracted by using a standardized data extraction framework. Two independent reviewers will conduct the data extraction, and they will discuss any discrepancies or disagreements with a third reviewer. A data extraction framework was developed to guide the extraction and charting process for the data from the articles. It will consist of standard bibliographical information (title, author, journal, year of publication, language, location of the study, and study objectives). Additional information, such as the type of study, the primary outcome, and other valuable information, will be included as well. The framework will provide an overview of the significant information about the studies and facilitate data analysis.

### Stage 5: Collating, Summarizing, and Reporting the Results

Our scoping review intends to present an overview of the study area, unlike a systematic review, which requires meta-synthesis reporting. Thus, we will report on the information we gather based on the selection criteria. Our study will summarize all of the data and information from relevant articles and will emphasize the scope of malaria vectors in Malaysia. Furthermore, gaps in research regarding specific study areas will be identified and determined. The study will use the PRISMA-ScR reporting guidelines to accurately report the scoping review and search results.

### Ethical Considerations

Ethical approval is not required, as our study does not involve collecting data from human participants.

### Dissemination

An article detailing the findings of the scoping review will be submitted to a scientific journal for publication. Study findings will be disseminated via open-access publications in peer-reviewed journals, presentations to stakeholders, relevant meetings, conferences, and continuous medical education at the department level, as well as future seminars and workshops. The published article will be included as part of a dossier for the Malaria Elimination Program of Ministry of Health Malaysia.

## Results

The literature search was conducted in March 2022, yielding 631 malaria vector–related articles. A total of 48 studies were found to be eligible after accessing and evaluating the articles. As of early 2022, our scoping review reached the data synthesis stage. Results are expected to be published by the end of 2022 in a peer-reviewed journal.

## Discussion

Malaria is incredibly harmful to very young children and low-income individuals [27]. The eradication of malaria is among the best returns on investments in public health and is very cost-effective [27]. In endemic nations, including Malaysia, initiatives for preventing and eventually eradicating malaria are increasingly seen as high-impact strategic investments that will benefit public health, reduce poverty, advance equity, and advance overall development [28].

Vector control is a crucial part of malaria prevention, control, and eradication programs due to its tremendous potential to improve personal protection and reduce disease transmission [29]. The ability of vectors to transmit parasites and their susceptibility to vector control techniques vary by mosquito species and are impacted by regional ecological parameters. Vector control must be implemented on the basis of local epidemiological and entomological data. Entomological surveillance and monitoring of the coverage and effects of vector control interventions must be included in national surveillance systems in order to provide an efficient vector control response [30].

The distribution of mosquitoes in Malaysia was last reviewed in 1997, with a focus on *Anopheles* mosquitoes, by Rahman et al [12], but their focus was not specifically on malaria vectors. Later, Vythilingam and Hii [13] outlined the malaria vectors in Malaysia, focusing on human malaria. The desired elimination of malaria is thought to be affected by extended delays in updating information on vectors. A systematic study conducted by Bertola et al [31] on the occurrence and bionomics of potential malaria vectors successfully supported the identification of 3 new potential vectors in Europe, along with a study conducted by Sinka et al [32]. Our scoping review will provide up-to-date insights on established and potential human and zoonotic malaria vectors, which we believe will contribute to the development of strategies for achieving malaria elimination in Malaysia. However, it is crucial that Malaysian researchers continue to study malaria vectors in order to make decisions about vector control measures that will help to eradicate the disease.

The lack of current information on malaria vectors in Malaysia indicates the need for more extensive research and the systematic compilation of entomological data. The development of effective malaria interventions, such as tools for selecting vector control options and strategies and managing vector control, as well as the acceleration of the certification of malaria eradication, can benefit from such information. However, a limitation of our study is the exclusion of any unpublished data, as we aim to ensure that the sources on which our findings are based are reputable by only including peer-reviewed studies in our scoping review.

Our novel scoping review of malaria vectors in Malaysia will provide a comprehensive evidence summary of the updated status of malaria vectors. An understanding of the status of *Anopheles* as malaria vectors and the knowledge generated from the behavioral characteristics of malaria vectors are the key components in making effective interventions for eliminating malaria.

## Abbreviations

PRISMA-ScR: Preferred Reporting Items for Systematic Reviews and Meta-Analyses Extension for Scoping Reviews
WHO: World Health Organization

## Data Availability

Data sharing is not applicable to this article, as no data sets have been generated or analyzed during the study at this time.
